# Is increased carer knowledge of the health care system associated with decreased preventable hospitalizations for people in the community diagnosed with dementia? A systematic review protocol

**DOI:** 10.1186/s13643-018-0875-6

**Published:** 2018-11-24

**Authors:** Jane V. Tehan, Anita Panayiotou, Helen Baxter, Paul Yates, Joanne Tropea, Frances Batchelor, Melbourne Ageing Research Collaboration

**Affiliations:** 1 Austin Health, Heidelberg, VIC Australia; 2 National Ageing Research Institute, Parkville, VIC Australia; 3 Melbourne EpiCentre, Melbourne Health and University of Melbourne, Parkville, VIC Australia

**Keywords:** Dementia, Carers, Hospitalization

## Abstract

**Background:**

People living with dementia (PLWD) are admitted to hospital twice as often as those without dementia, for ambulatory care sensitive conditions (ACSC) that could have been managed in ambulatory and primary care settings. PLWD are at greater risk of poor outcomes during and following hospital admission. Compared to those without dementia, they are almost twice as likely to die in hospital and two to three times more likely to experience an adverse event. Although some hospitalizations are clinically necessary, there may be a proportion related to ACSC that could be potentially avoided with additional support and education for PLWD and their carers. This study aims to assess the effectiveness of interventions focused on reducing avoidable hospitalization for PLWD by supporting carers to manage the health care needs of the PLWD, via improved awareness and understanding of health and the healthcare system.

**Method:**

Scientific and gray literature will be searched using a combination of keywords pertaining to dementia, caregivers, education, and support. Included studies will involve community-dwelling PLWD and caregivers, with interventions aimed at improving carer’s understanding of the healthcare system and ability to manage the caregiving role. The primary outcome will be hospitalization related to the PLWD and secondary outcomes will be carer burden, stress, wellbeing, and quality of life. All study designs will be considered. Data from included studies will be analyzed using descriptive statistics and content analysis. If the data permits, we will perform a meta-analysis and subgroup analyses, related to the intervention and participant characteristics.

**Discussion:**

This review will provide a comprehensive picture of the knowledge available on the subject and identify knowledge gaps in existing literature. The findings may highlight the lack of existing interventions for PLWD and their carers who live in the community and will help stakeholders to identify needs and develop programs targeted to carers and care recipients that prevent avoidable hospitalization for PLWD.

**Systematic review registration:**

PROSPERO number: 49655.

## Background

There are approximately 354,000 Australians living with dementia, and it is the single greatest cause of disability in older adults (65 years or over) [1, 2]. Dementia affects 50 million people worldwide, with prevalence rates expected to triple by 2050 [3].

People living with dementia (PLWD) are major users of acute care services. Studies examining the overrepresentation of PLWD in hospital have found more than 20% of inpatients aged 70 or older had dementia [4]. PLWD are admitted to hospital twice as often as those without dementia, for ambulatory care sensitive conditions (ACSC) that could otherwise have been managed in ambulatory and primary care settings [4]. The increased demand on the hospital system for PLWD is evident for all cause admissions, controlling for age, sex, and comorbidity.

This growing issue has an impact not only on PLWD but families and more broadly on society. PLWD are at greater risk of poor outcomes during and following hospital admission. They are almost twice as likely to die in hospital. Compared to those without dementia, PLWD are two to three times more likely to experience an adverse event, such as falls resulting in fracture, delirium, sepsis, or pressure ulcers, during their hospital admission [5–8]. The hospital environment and particularly the emergency department (ED) can have deleterious impacts on PLWD. Many hospitals do not have appropriately trained staff to manage the complexities of providing healthcare for PLWD within the general ward setting [7]. Emergency departments often have high levels of activity and stimulation, which can cause or exacerbate confusion and agitation in PWLD; dementia is under-detected; and staff lack knowledge and expertise in relation to dementia [8]. Additionally, carers and families are placed under increased stress.

There are clear societal implications too, especially with the greater burden on health care systems. The average hospital cost for PLWD is higher than in those without, irrespective of the principal diagnosis [8]. Once admitted, the median length of stay is longer for those with a diagnosis of dementia for most conditions [8]. Length of stay makes up a large proportion of the total health cost for an admission.

Although some presentations to hospital are clinically necessary, there may be a proportion of presentations and admissions related to ACSC including carer stress, escalation in challenging behaviors, delirium, and continence or wound management which could be potentially avoided with additional support and education for PLWD and their carers.

There are a number of strategies currently being explored to prevent avoidable hospitalizations for PLWD in residential aged care. However, in Australia, the majority (70%) of PLWD reside in the community [9]. Ninety-two percent of these individuals receive support from family and friends, with a national estimate of 200,000 informal carers in the community [2]. Despite this significant number, there are no formal guidelines or recommendations designed for carers, aimed at preventing avoidable hospitalization.

A systematic review by Phelan et al. [10] aimed to identify interventions that prevent hospitalization for those in the community with dementia. The available literature until December 2013 was reviewed with 10 studies identified for inclusion based on the following criteria: (1) published in English, (2) included a control or comparison group, (3) reported hospitalization, and (4) included community-dwelling older adults with dementia. Most of the intervention strategies of the included studies consisted of face-to-face assessments of PLWD and their caregivers and the development and implementation of a care plan. None of the studies found a significant reduction in hospital admissions. Five years on, there has been growing interest and several newer publications on this topic. In the current systematic review, the selection criteria were broadened to those of Phelan et al. and will evaluate several additional publications that substantially add to the knowledge on this topic.

### Objectives

The study aims to assess the effectiveness of interventions aimed at reducing avoidable hospitalization for PLWD by supporting carers to manage the health care needs of the PLWD, via improved awareness and understanding of health and the healthcare system.

### Question

How effective are interventions at (1) improving carers’ understanding of healthcare decision making and (2) preventing avoidable hospital presentations for community-dwelling PLWD?

## Methods

Refer to the PRISMA-P checklist for details.

### Protocol and registration

The current systematic review protocol has been registered on PROSPERO number: 49655 https://www.crd.york.ac.uk/prospero/display_record.php?RecordID=49655

### Eligibility criteria

#### Participants/population

The participant group is the PLWD and carer dyad. Caregivers may be informal and unpaid (i.e., family members, friends, or community members) or paid employees, adults (18 years of age or older). They must provide support in one or more activity of daily living and may either reside with the care recipient or reside separately. The care recipient must live in the community and have a formal diagnosis of dementia.

#### Interventions

The interventions of the study are any interventions targeted at carers including formal resources, training, or support, additional to information or assistance already freely available, aimed at improving the carer’s understanding of the healthcare system and their ability to manage the health-related aspects of the caregiving role (e.g., which healthcare services to access, encouraging regular GP appointments). Interventions may be delivered in a hospital, primary care, or community setting but not in institutionalized care.

#### Comparisons

The comparison group included those not receiving any active intervention or any other type of active intervention(s) beyond usual care.

### Outcomes

#### Primary outcome

The primary outcome includes hospital admissions or presentations related to the PLWD.

#### Secondary outcomes

We will also focus on validated measures of carer burden, stress, wellbeing, or quality of life, including questionnaires such as the Zarit Burden Interview, the Depression Anxiety and Stress Scale or the Hospital Anxiety and Depression Scale, and the Health Status Questionnaire.

#### Study designs

Randomized controlled trials, cluster randomized controlled trials, before and after studies, and observational studies will be included. Systematic literature reviews and narrative reviews will be reviewed with their reference lists examined. The TIDieR checklist [11] will be used to describe the intervention included in each trial and to improve comparability between studies.

### Inclusion criteria

Studies focused on evaluating interventions for people living in the community with a formal diagnosis of dementia and their carers, with an outcome relating to hospital presentations. All study designs will be considered. We will include studies published in all languages and contact authors for unpublished data wherever it appears relevant.

### Exclusion criteria

Studies involving interventions structured as a model of care (e.g., Hospital Admission Risk Program (HARP), Immediate Response Service) will be excluded as they are beyond the scope of a single targeted intervention. Studies that were conducted in a residential aged care setting will be excluded.

### Search strategy

#### Information sources

An electronic database search will be performed using MEDLINE, PsycINFO, Embase, and Emcare via Ovid. Clinical trial registers will also be reviewed using the Cochrane Library Central Register of Controlled Trials (CENTRAL) and ClinicalTrials.gov. A search for gray literature will be performed using Trove, Google Advanced, Open Grey, ALOIS, and websites of relevant local organizations such as Alzheimer’s Australia (now Dementia Australia), Australian Institute of Health and Welfare, and the National Health and Medical Research Council. Manual searches of the reference lists of all included studies and reviews will also be performed to locate additional relevant studies.

### Search

The full electronic search strategy that will be used for the retrieval of citations from MEDLINE via the Ovid Platform is presented in Table 1.

**Table 1:** Full electronic search strategy for retrieval of citations from MEDLINE via the Ovid Platform

1	exp Dementia/
2	dement*.mp.
3	alzheimer*.mp.
4	(lewy* adj2 bod*).mp.
5	(chronic adj2 cerebrovascular).mp
6	(organic brain disease or organic brain syndrome).mp.
7	(cerebr* adj2 deteriorat*).mp.
8	(cerebral* adj2 insufficient*).mp.
9	or/1-8
10	caregivers/
11	(carer or caregiver* or care giver* or family or families or spouse or parent or kin or relatives or daughter or son or partner or husband or wife or neighbo* or friend*).mp.
12	10 or 11
13	Health education/ or consumer health information/ or Health literacy/ or patient education as topic/ or health promotion/ or health behavior/ or health knowledge, attitudes, practice/ or health services for the aged/ or evidence-based practice/
14	(psychoeducation or health literacy or evidence based program* or health promotion).mp.
15	(carer or caregiver or care giver) adj3 (information or intervention or counselling or counseling or support or education or program*)).mp.
16	13 or 14 or 15
17	9 and 12 and 16

### Data collection and analysis

#### Study selection

After performing the electronic searches, duplicates will be removed. Two review authors (AP, JVT) will independently screen the titles and abstracts in Covidence systematic review software, Veritas Health Innovation, Melbourne, Australia (available at www.covidence.org), to identify studies that meet the inclusion criteria. Any disagreement will be resolved by discussion or, if needed, by consulting a third reviewer (either FB or PY). Full-text articles will be reviewed by AP and JVT for inclusion eligibility. The reasons for exclusion will be reported.

### Data extraction and management

Two review authors (AP, JVT) will independently perform data extraction using a standardized data collection form in Covidence. In case of disagreement, a third review author (either FB or PY) will be consulted to reach a consensus. For each study, data extraction and reporting will include the following: trial registry identification, funding and potential conflicts of interest, main methodological characteristics, outcomes assessed, and results. Using the TIDieR checklist [11], the main characteristics of each trial will be described, paying special attention to the interventions assessed.

### Data items

Data from included studies will be analyzed using descriptive statistics and content analysis. Outlined in Table 2, study characteristics (e.g., year of publication, study population) will be collected, along with the intervention characteristics (e.g., duration, delivery mode, content, setting/location).

**Table 2:** Data items to be extracted from each included study

Study characteristics	Year of publication
	Sample (*n*, age)
	Country
	Study design
Study population	Caregiver relationship to care recipient (e.g., spouse, child, friend)
	Caregiver characteristics (e.g., paid or unpaid, cultural background)
	Dementia diagnosis (e.g., type, severity, onset)
	Living arrangement (e.g., alone, with spouse or other, urban or rural setting)
Intervention characteristics	Duration
	Mode of delivery (e.g., face to face, online, group, individual)
	Content (e.g., psychoeducation, decision trees)
	Location and setting (e.g., GP clinic, hospital)
Hospital outcome measures	Hospital admissions/presentations (e.g., number, type, length of hospital stay, reasons based on primary and secondary discharge diagnoses, including whether they met the definition of ACSC according to [13])
	Adverse incidents (e.g., type, frequency)
Carer outcome measures	Carer burden/stress/wellbeing/quality of life (e.g., Zarit Burden Interview, Depression Anxiety and Stress Scale, Hospital Anxiety and Depression Scale, Health Status Questionnaire)

### Risk of bias in included studies

Two review authors (AP, JVT) will independently assess the risk of bias in included studies by using the Cochrane risk of bias tool for randomized controlled trials and the CASP criteria for all other study types. Any disagreement will be resolved by discussion or, if needed, by requesting a third review author to also rate the studies (either FB or PY). The following sources of bias will be assessed: selection bias (including random sequence generation and allocation concealment), performance bias (blinding of participants and personnel), detection bias (blinding of outcome assessments), attrition bias (incomplete outcome data), and reporting bias (selective reporting). Risk of bias will be reported as either “low risk,” “unclear risk,” or “high risk,” and an explanation for each rating will be provided.

### Summary measures

The measure of treatment effects across studies will be risk ratios with a 95% confidence interval (CI) for dichotomous variables and mean difference with a 95% CI for continuous variables. Standardized mean difference with its 95% CI will only be used if similar outcome constructs are measured with different rating scales.

### Unit of analysis issues

The unit of analysis of interest will be the individual allocated to the intervention or comparison groups in the included trials. If cluster randomized trials are found, we will follow the methods recommended in the Cochrane Handbook for Systematic Reviews of Interventions [12].

### Dealing with missing data

Where there is missing data, we will contact the corresponding author of the study.

### Assessment of heterogeneity

Each reported comparison will include an assessment of between-studies heterogeneity using the *I*^2^ statistic for meta-analysis. We will combine the results of the studies if we consider it meaningful to do so and if the *I*^2^ statistic values are moderate (> 60%). Otherwise, we will not combine the studies and will only provide subgroup analysis or individual results.

### Assessment of reporting bias

If the required number of 10 studies is reached for an outcome, we will perform an assessment of the risk of reporting bias using funnel plots. We will also make contact with authors to request results if we find any clinical trial protocols that are not published and appear to be completed.

### Data synthesis

The anticipated heterogeneity between the included studies, both with regard to diversity of interventions and outcomes measured, may make a quantitative pooling of the outcome data inappropriate. In this case, we will present a narrative synthesis of the different interventions identified, along with the main results of the included studies. If the between-studies heterogeneity permits, we will perform a meta-analysis for each comparison that includes at least two studies, using a random effects model. Review Manager (RevMan) version 5 will be used for the analysis.

### Risk of bias across studies

Two review authors (AP, JVT) will independently assess the quality of evidence using the GRADE approach for each outcome measure. Any disagreement will be resolved by discussion. The following sources of bias will be assessed: imprecision, inconsistency, indirectness, risk of bias, and publication bias.

### Subgroup analysis and investigation of heterogeneity

Depending on the between-studies heterogeneity and the availability of sufficient data, we will conduct subgroup analyses according to:

- The intensity/length of the intervention provided;
- The application format of the intervention (individual- versus group-based);
- The characteristics of the caregivers (e.g., cultural and linguistic background, rural versus urban setting);
- The characteristics of the carer recipient (e.g., severity of dementia, type of dementia, rural versus urban setting).

## Discussion

This review will provide a comprehensive picture of the knowledge available on the subject and identify knowledge gaps in existing literature. The findings may highlight the lack of existing interventions for PLWD and their carers who live in the community and will help stakeholders to identify needs and develop programs targeted to carers and care recipients that prevent avoidable hospitalization for PLWD.

### Limitations

Some difficulties may impact on the completion and objectives of this study. This includes the potential for the included studies (and reported data) to be too few to address the research questions. However, this is not uncommon for systematic reviews and may encourage future research addressing this gap. In addition, there may be variability in the definition of the main concepts (e.g., dementia), and these variations could affect comparisons between the included studies. To address this and enable data analysis, definitions and measures used by each study for main concepts will be detailed when conducting the data extraction. Lastly, there is the potential that the data retrieved from different studies may not be applicable to every country due to differences in the healthcare system.

## Abbreviations

ACSC: Ambulatory care sensitive conditions
PLWD: People living with dementia
